# Nationwide epidemiological study of severe gallstone disease in Taiwan

**DOI:** 10.1186/1471-230X-9-63

**Published:** 2009-08-22

**Authors:** John Huang, Chia-Hsuin Chang, Juin-Ling Wang, Hsu-Ko Kuo, Jou-Wei Lin, Wen-Yi Shau, Po-Huang Lee

**Affiliations:** 1 Department of Surgery, National Taiwan University Hospital, Taipei, Taiwan, Republic of China; 2 Department of Internal Medicine, National Taiwan University Hospital, Taipei, Taiwan, Republic of China; 3 E-Da Hospital, I-Sho University, Kaohsiung County, Taiwan, Republic of China; 4 Department of Internal Medicine, National Taiwan University Hospital, Yun-Lin Branch, Douliou City, Taiwan, Republic of China; 5 Division of Health Technology Assessment, Center for Drug Evaluation, Taipei, Taiwan, Republic of China

## Abstract

**Background:**

Our study aimed to assess the nationwide trends in the incidence of severe gallstone disease in Taiwan among adults aged ≥20.

**Methods:**

A retrospective longitudinal study was conducted using Taiwan National Health Insurance Research Database collected during 1997–2005. Patients with incident severe gallstone disease (acute cholecystitis, biliary pancreatitis, acute cholangitis) and gallstone-related procedures (elective and non-elective cholecystectomy, endoscopic retrograde cholangiopancreatography [ERCP]) that led to hospital admission were identified using ICD-9-CM diagnostic and procedure codes. Annual incidence rates of gallstone-related complications and procedures were calculated and their 95% confidence intervals (CI) were estimated assuming a Poisson distribution.

**Results:**

The hospital admission rate for severe gallstone disease increased with advancing age and the age-standardized rate (95% CI) per 1000 population was 0.60 (0.59–0.60) for men and 0.59 (0.59–0.60) for women. Men had a higher rate of acute cholecystitis, probably due to the substantially lower rate of elective cholecystectomy among men than women. For those aged 20–39, hospital admissions for all gallstone-related complications and procedures increased significantly. For those aged ≥60, incidences of biliary pancreatitis, acute cholangitis, and hospital admission for gallstone receiving ERCP increased significantly without substantial change in the incidence of acute cholecystitis and despite a decreased rate of elective cholecystectomy.

**Conclusion:**

This population-based study found a substantial increase in the rate of admission for severe gallstone disease among those aged 20–39. Concurrently, the incidences of biliary pancreatitis and acute cholangitis have risen among those aged ≥60.

## Background

Gallstone disease was once considered a disease of the West [1]. In the US, it is estimated to affect 20 – 25 million adults (i.e., 10–15% of the population) [2,3]. Of the approximately one third of individuals with gallstones who develop symptoms, 80% experience biliary colic [4]. Gallstone disease and its complications (cholecystitis, pancreatitis, and cholangitis) are the major causes of gastrointestinal morbidity that lead to hospital admission [5]. According to the National Hospital Discharge Survey, cholelithiasis led to 325,000 hospitalizations in the US in 2005 [6]. The estimated total number of cholecystectomies, including laparoscopic procedures, numbered as high as 770,000 in 1996, and was associated with an expense of more than 2 billion US dollars [7,8]. In the UK, approximately 5.5 million people have gallstones and over 50,000 cholecystectomies are performed each year [5].

The prevalence of gallstone disease is higher in older individuals and females. Several sonographic screening studies conducted in China, Taiwan, Japan, and Korea found gallstone disease in 5–10% of the population, which is about half of rate found in the West [9-14]. Clinically, the incidence of gallstone disease has been increasing in the past decade coincident with the rise in calorie and fat consumption, decrease in fiber intake, and increased prevalence of the sedentary lifestyle in the Asian population [15]. However, estimates of the population-based incidence of symptomatic gallstone disease and related complications are lacking. We analyzed Taiwan National Health Insurance Research Database to assess the ten-year trend in symptomatic gallstone disease and related complications requiring hospitalization among different gender and age groups.

## Methods

### Data source

The protocol of this study was approved by the National Taiwan University Hospital Research Ethics Committee, Taipei, Taiwan. A universal compulsory national health insurance program was launched by the Taiwanese government in March 1995. By the end of 2005, approximately 98% of the population was insured [16]. Large computerized administrative and claims datasets compiled by the National Health Research Institutes, Taiwan, are made available to investigators for research purposes after individual health information is de-identified. In this study we used the inpatient claims dataset. The source population for this study was all beneficiaries aged 20 or above during 1996–2005.

### Selection of cases

Severe gallstone disease was defined as gallstone-related complications (acute cholecystitis, biliary pancreatitis, and acute cholangitis) that lead to hospitalization, as well as symptomatic disease requiring surgical or endoscopic procedures, which included elective and non-elective cholecystectomy (depending on whether patients had acute cholecystitis preoperatively) and endoscopic retrograde cholangiopancreatography (ERCP). Asymptomatic disease, disease of lesser severity that did not result in hospitalization, and biliary colic not requiring surgical or endoscopic treatment was not studied. The Taiwan National Health Insurance Research Database during the period 1996–2005 was searched to identify potential cases from the populations at risk (i.e., hospitalized patients with a principal discharge diagnosis of cholelithiasis [The International Classification of Diseases, 9th Revision, Clinical Modification, ICD-9-CM code 574] and patients admitted due to gallstone-related complications and procedures; Table 1). To reduce the likelihood of misdiagnoses, we excluded anyone diagnosed as having primary or secondary liver cancer (155), cancer of the gallbladder and extrahepatic bile ducts (156), pancreatic cancer (157), gastric cancer (151), malignant neoplasm of other sites (140–150, 152–154, 158–199), and hematological malignancy (200–208). Those with acquired immunodeficiency syndrome or human immunodeficiency virus infection (V08, 042), and those with unavailable or incorrect age and gender information were also excluded. The incidence of biliary pancreatitis was calculated after excluding cases possibly caused by alcohol use (ICD-9-CM codes for abuse or intoxication 303.0 and 303.9).

**Table 1:** Definition of gallstone-related complications and procedures based on the ICD-9-CM codes

Categories	ICD-9-CM codes
Acute cholecystitis	574.0, 574.3, 574.6, 574.8

Biliary pancreatitis	574 plus 577.0, 577.1

Acute cholangitis	574 plus 576.1

Elective cholecystectomy	574, 574.1, 574.2, 574.4, 574.5, 574.7, 574.9 plus 51.22, 51.23

Non-elective cholecystectomy	574.0, 574.3, 574.6, 574.8 plus 51.22, 51.23

Gallstones receiving ERCP	574 plus 51.10, 51.11, 51.64, 51.84, 51.85, 51.86, 51.87, 51.88, 52.13

Endoscopic retrograde cholangiopancreatography

A study patient could have one or more hospitalizations during the study period. An incident event was counted if the patient had no related hospital discharge diagnoses in the claims database prior to admission. We excluded data in 1996 because events occurred in that year that could not be correctly counted as incident or prevalent events.

### Data collection

The data included the patient's age, gender, hospitalization year, and comorbidities (i.e., diabetes mellitus [ICD-9-CM code 250], hyperlipidemia [272.0–272.4], chronic liver diseases [571], alcoholism [303.0, 303.9], and hereditary or acquired hemolytic anemia [282, 283]).

### Statistical analysis

We calculated the gender distribution, age distribution, and proportion of patients with comorbidities for all cases during 1997–2005. Annual rates of hospital admission for severe gallstone disease (acute cholecystitis, biliary pancreatitis, acute cholangitis), individual complications, and gallstone-related procedures (elective and non-elective cholecystectomy, ERCP) among the study population were calculated by dividing the number of patients with confirmed incident gallstone disease by the mid-year Taiwanese population of that year. The calculation of 95% confidence intervals assumed Poisson distribution of the number of cases. The age- and gender-specific annual incidence rates were calculated for groups aged 20–39, 40–59, 60–79, and ≥80 years. Age-adjusted gender-specific rates were directly standardized by applying the age-specific rates to those of the population in 2000.

If the data series exhibited an upward or downward trend during the study period, we examined whether the confidence intervals in the first and last year overlapped. If the confidence intervals did not overlap, a significant increase or decrease in the rate was concluded and the percent change in the incident rate between 1997 and 2005 was calculated. To examine the annual trend in incidence rates for men and women during 1997–2005, age-adjusted Poisson regression analysis was carried out using calendar year as an ordinal variable. Likelihood ratio test was used to compare the models with and without gender-by-year interaction terms to test the difference in trends between men and women. Two-sided *p *value < 0.05 was considered to be statistically significant.

#### Ethical approval

National Taiwan University Hospital Research Ethics Committee.

## Results

Totally, 273,385 adults aged ≥20 were hospitalized for all gallstone diseases and related complications during 1997–2005, but only 208,516 fulfilled the inclusion criteria of severe gallstone disease. Among them, 170,781 were identified with incident hospital admissions. After excluding patients with primary and metastatic cancer of the liver, pancreas, and bile duct (n = 5377); malignant neoplasm of other sites (n = 7826); hematological malignancy (n = 441); human immunodeficient virus infection/acquired immunodeficiency syndrome (n = 9); and unavailable or incorrect gender or age information in the database (n = 800), 155,322 incident cases (71,187 [45.8%] men and 84,135 [54.2%] women) were included in our study. Concomitant diagnoses included diabetes in ~11% of cases, chronic liver disease in 3%, hyperlipidemia in 1%, alcoholism in 0.2%, and hemolytic disease in 0.3%. The mean annual population of Taiwan aged 20 years or older during 1997–2005 was 15,863,966. Table 2 summarizes the average rates of hospital admissions for severe gallstone disease, gallstone-related complications, and gallstone-related procedures for men and women in the overall population and each age group.

**Table 2:** Average incident hospital admission rates for all severe gallstone disease, related complications and procedures among different age groups and overall in Taiwan per 1,000 population 1997–2005

	Men		Women	
	Number of admissions	Rate per 1,000 population (95% CI)	Number of admissions	Rate per 1,000 population (95% CI)

**Severe gallstone diseases**				

20 – 39	4,654	0.13 (0.13–0.14)	5,250	0.16 (0.15–0.16)

40 – 59	13,831	0.55 (0.54–0.56)	13,125	0.53 (0.52–0.54)

60 – 79	20,037	1.80 (1.77–1.82)	18,245	1.74 (1.71–1.76)

≥80	4,712	3.28 (3.18–3.37)	5,178	3.40 (3.30–3.49)

All ages	43,234	0.60 (0.59–0.60)	41,798	0.59 (0.59–0.60)

**Gallstone-related complications**				

Acute cholecystitis				

20 – 39	3,717	0.11 (0.10–0.11)	4,214	0.13 (0.12–0.13)

40 – 59	10,697	0.43 (0.42–0.43)	9,645	0.39 (0.38–0.40)

60 – 79	15,418	1.38 (1.36–1.40)	13,443	1.28 (1.26–1.30)

≥80	3,488	2.43 (2.35–2.51)	3,804	2.50 (2.42–2.57)

All ages	33,320	0.46 (0.46–0.46)	31,106	0.44 (0.44–0.45)

Biliary pancreatitis				

20 – 39	938	0.03 (0.03–0.03)	869	0.03 (0.02–0.03)

40 – 59	2,420	0.10 (0.09–0.10)	2,219	0.09 (0.09–0.09)

60 – 79	2,869	0.26 (0.25–0.27)	3,123	0.30 (0.29–0.31)

≥80	654	0.45 (0.42–0.49)	786	0.52 (0.48–0.55)

All ages	6,881	0.10 (0.09–0.10)	6,997	0.10 (0.10–0.10)

Acute cholangitis				

20 – 39	375	0.01 (0.01–0.01)	529	0.02 (0.01–0.02)

40 – 59	2,087	0.08 (0.08–0.09)	2,479	0.10 (0.10–0.10)

60 – 79	4,183	0.38 (0.36–0.39)	3,905	0.37 (0.36–0.38)

≥80	1,273	0.89 (0.84–0.93)	1,354	0.89 (0.84–0.94)

All ages	7,918	0.11 (0.11–0.11)	8,267	0.12 (0.11–0.12)

**Gallstone-related procedures**				

Elective cholecystectomy				

20 – 39	4,771	0.14 (0.13–0.14)	9,395	0.28 (0.28–0.29)

40 – 59	11,915	0.47 (0.47–0.48)	19,861	0.80 (0.79–0.81)

60 – 79	12,963	1.16 (1.14–1.18)	15,414	1.47 (1.44–1.49)

≥80	1,793	1.25 (1.19–1.30)	1,775	1.16 (1.11–1.22)

All ages	31,442	0.43 (0.43–0.44)	46,445	0.66 (0.65–0.67)

Non-elective cholecystectomy				

20 – 39	2,791	0.08 (0.08–0.08)	3,606	0.11 (0.10–0.11)

40 – 59	8,369	0.33 (0.33–0.34)	7,919	0.32 (0.31–0.33)

60 – 79	12,327	1.11 (1.09–1.13)	10,385	0.99 (0.97–1.01)

≥80	2,378	1.65 (1.59–1.72)	2,197	1.44 (1.38–1.50)

All ages	25,865	0.36 (0.35–0.36)	24,107	0.34 (0.34–0.35)

Gallstones receiving ERCP				

20 – 39	1,244	0.04 (0.03–0.04)	1,674	0.05 (0.05–0.05)

40 – 59	4,561	0.18 (0.18–0.19)	4,880	0.20 (0.19–0.20)

60 – 79	8,296	0.74 (0.73–0.76)	7,684	0.73 (0.72–0.75)

≥80	2,163	1.50 (1.44–1.57)	2,156	1.41 (1.35–1.47)

All ages	16,264	0.22 (0.22–0.23)	16,394	0.23 (0.23–0.24)

ERCP: endoscopic retrograde cholangiopancreatography

### Age-standardized trend of gallstone-related complications

**Figure 1:**
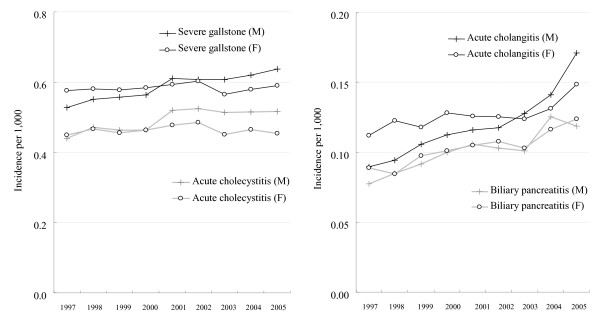
**Age-standardized incident hospital admission rates of severe gallstone stone disease and related complications for men and women from 1997 to 2005**.

Figure 1 shows trends in the age-standardized incidence rates of gallstone-related complications during 1997–2005. Women had relatively stable incidence of severe gallstone disease and acute cholecystitis, whereas men had significantly increased incidence of severe gallstone disease and all related complications (test for trend *p *< 0.001). Furthermore, the increase in the incidence of acute cholangitis was more marked for men (91%) than for women (32%; test for interaction *p *< 0.001). The increase in the incidence of biliary pancreatitis was similar for men and women (test for interaction *p *= 0.07).

**Figure 2:**
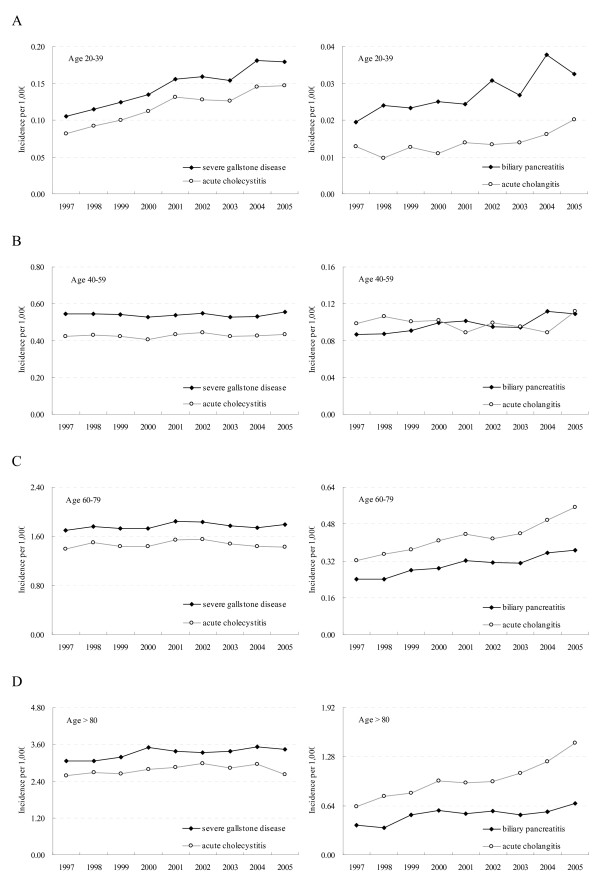
**Hospital admission incident trends of gallstone-related complications for age group (A) 20–39 (B) 40–59 (C) 60–79 (D) ≥80 during 1997–2005**.

Figure 2 shows the trends in the incidence of severe gallstone disease and complications among different age groups (20–39, 40–59, 60–79, and ≥80). In the younger population (aged 20–39), increase in the incidences of all gallstone-related complications was significant (82% for acute cholecystitis, 70% for biliary pancreatitis, and 63% for acute cholangitis; test for trend *p *values < 0.001) (Fig. 2A). In the elderly population (aged ≥60), the incidence increase was small for acute cholecystitis but significant for acute cholangitis and biliary pancreatitis, especially among those aged ≥80 (*p *values < 0.001) (Fig. 2D). The mean age of patients with gallstone complications after 60 years increased significantly during the study period from 71.6 to 73.5 year-old for men and 71.9 to 73.7 year-old for women (test for trend *p *values < 0.001). For the age group 40–59, the incidence of all gallstone-related complications remained stable during the study period (Fig. 2B).

### Age-standardized trend of gallstone-related procedures

**Figure 3:**
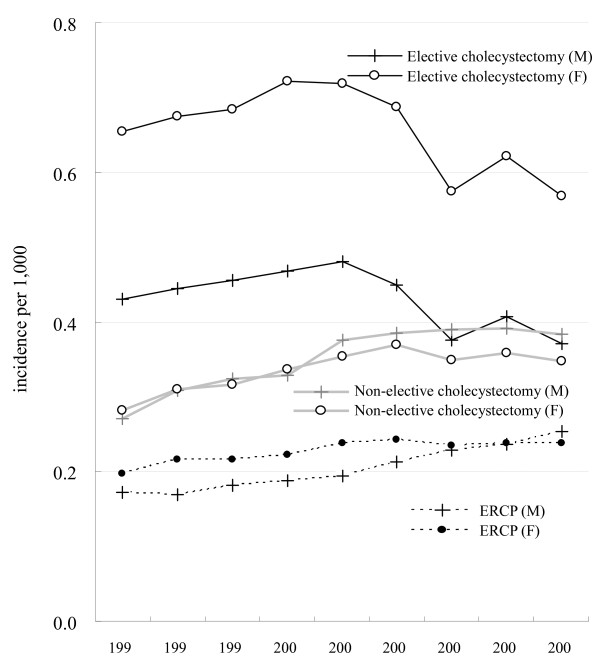
**Age-standardized hospital admission rates of gallstone-related procedures for men and women from 1997 to 2005**.

**Figure 4:**
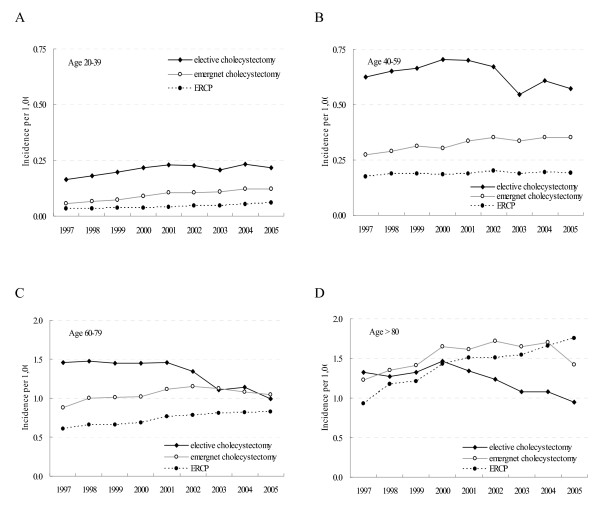
**Trends in incident hospital admission rate of gallstone-related procedures for age group (A) 20–39 (B) 40–59 (C) 60–79 (D) ≥80 during 1997–2005**.

Women had significantly higher rates of elective cholecystectomy than men (Fig. 3). For both men and women, the hospital admission rates for elective cholecystectomy increased steadily from 1997 to 2001 then gradually decreased especially during the Severe Acute Respiratory Syndrome outbreak in 2003, with the most marked decline occurring among those aged 60–79 and ≥80 (Fig. 4C, 4D). In contrast, for the younger population (aged 20–39), hospital admissions for elective cholecystectomy rose steadily during 1997–2001 and then became plateau (Fig. 4A). Also, they had a continuing increasing rate of non-elective cholecystectomy. For both men and women, the upward trend in hospital admissions for gallstone receiving ERCP was significant (test for trend *p *< 0.001). The increasing use of ERCP occurred in almost all age groups with the most striking increase occurring among those aged ≥80.

## Discussion

In this study, an analysis of national health insurance claims data found that the rate of hospital admission for severe gallstone disease per 1000 population in Taiwan was 0.60 for men and 0.59 for women during 1997–2005. Furthermore, hospital admissions for all gallstone-related complications and procedures were significantly increased among those aged 20–39. Incidence of biliary pancreatitis and acute cholangitis and hospital admission for ERCP increased significantly among those aged ≥60 years.

Several population-based studies in England, Northern Europe, Canada, and the US have observed an increase in hospital admission and operation rate for gallstone diseases since the 1950s, followed by a modest decrease in the 1980s and a substantial increase in the 1990s after the introduction of laparoscopic cholecystectomy [17-20]. Kang and colleagues reported a steady increase in hospitalization rate for gallstone disease in England from 68.7 to 104.9 per 100,000 population between 1989/1990 and 1999/2000, although the proportion of patients receiving an operation fell significantly by 16.9% during the study period [21]. Urbach and Stukel examined the trends in incidence of gallstone disease and related complications that lead to hospitalization in Canada. They found the incidence rate of all severe gallstone diseases per 100,000 population decreased from 127.8 in 1988–1991 to 114.2 in 1992–2000 [22] and attributed this decline to the widespread adoption of laparoscopic cholecystectomy leading to a reduction in the incidence of acute cholecystitis.

The present study findings, in general, support our clinical observation that the rate of hospital admission for severe gallstone disease has been increasing in Taiwan during the past decade. It seems to be related in part to the increased accessibility of the patients to hospitals after changing the insurance system in the country although a modest increase in incidence of gallstone diseases in 10-year intervals might also play a role. Especially striking is the increase in the incidence of acute cholecystitis and hospital admission for elective cholecystectomy among those aged 20–39. This rise is mainly due to improved sonographic methods and thereby earlier diagnosis and may be due to a lowered threshold for laparoscopic surgery. Meanwhile, several studies have reported an association of obesity, decreasing intake of dietary fiber, higher intake of carbohydrate and fat, and less exercise with gallstone diseases, and the younger population might be particularly influenced by these factors [23-29].

The incidence of biliary pancreatitis per 100,000 population was 16.2 for men and 18.0 for women in Canada during 1988–2000 and 8.4 for men and 11.6 for women in Sweden during 1990–1994 [22,30]. Epidemiological studies suggested that bile duct stones are more likely to cause biliary pancreatitis and acute cholangitis in East Asia than in the western countries [31,32]. In the present study in Taiwan, the incidence of acute cholangitis was substantially higher than that in Canada, while the incidence of biliary pancreatitis was lower than that in Canada but close to that in Sweden. A systematic review of published articles about the epidemiology of acute pancreatitis in Europe found the incidence of acute biliary pancreatitis increased from 1970 to 2000 [33]. Several studies evaluating admissions for biliary surgery suggested that the proportion of patients with common bile duct stones decreased in Taiwan and Korea from 1980 to late 1990 [34-36]. Our study observed a significant increase in the incidence of biliary pancreatitis and acute cholangitis as well as in hospital admissions for ERCP especially among the elderly between 1997 and 2005. The finding that the mean age of the patients with complications after 60 years increased during the study period suggested this rise in incidence might be related to an increased segment of this elderly population. Although increased vigilance and improved diagnosis contributed substantially to this rise, other factors such as change in stone size or composition and preserved gallbladder motility due to delayed cholecystectomy might also have been contributors [37,38]. Further studies are required to evaluate the factors associated with the rise in the incidences of biliary pancreatitis and cholangitis.

Limited population-based data are available to examine the incidence of gallstone disease in East Asia. Several community- and hospital-based sonographic surveys observed an increase in gallstone prevalence with increasing age [9-14,34]. The prevalence of gallstone disease among asymptomatic participants was 0.7–1.5% for those aged <40; 2.6% for those aged 40–49; 6–8% for those aged 50–59; 11.9% for those aged 60–69; as high as 10–16% for those aged ≥65 years. Our findings were consistent with prior study results suggesting that incidence of severe gallstone disease leading to hospitalization is highest in the elderly. In Taiwan where pigmented stones are more prevalent, no predominance of gallstone disease in females was seen, as is common in western countries [1,30,31]. Women, though, did have a higher rate of elective cholecystectomy, probably because they were more likely to develop symptoms attributable to gallbladder stones [39,40]. The rate of acute cholecystitis was higher for men than women. However, this can not be entirely explained by the substantial lower rate of elective cholecystectomy rates in men versus women because the change in cholecystectomy rates were almost parallel in both genders during the period studied.

The strengths of our study were use of a nationwide data with large sample size, presentation of incidence trends in the recent decade, and attention to all gallstone-related complications and procedures. However, it also had several limitations. First, in this study as in several large database studies conducted in the UK, US, and Canada, patients with severe gallstone disease were identified by ICD-9-CM codes. Consequently, we could not exclude the possibility of an increasing tendency to code biliary colic as acute cholecystitis among younger patients. However, it is less likely because the incidence of elective and non-elective cholecystectomy as well as admissions for ERCP tended to increase in this age group. Second, the proportion of patients with comorbidities was likely to be under-estimated because these were probably under-reported in the discharge diagnoses. Third, the outcome of interest in this study was hospitalization for severe gallstone disease and related complications. Patients visiting the emergency department were not counted due to lack of data from ambulatory care records. And finally trends relating to gallstone location and composition could not be analyzed because this information was not available in the database.

## Conclusion

In conclusion, this population-based study observed a significant increasing trend in hospital admission rate for severe gallstone disease among those aged 20–39. Meanwhile, there has been a rise in the incidences of biliary pancreatitis and acute cholangitis among those aged ≥60.

## Competing interests

The authors declare that they have no competing interests.

## Authors' contributions

JH, JLW, CHC, and WYS designed the study and wrote the manuscript. HKK LPH and JWL gave critical suggestions on the study design, data analysis and manuscript revision. CHC reviewed the literature and undertook the analyses. All authors read and approved the final manuscript.

## Pre-publication history

The pre-publication history for this paper can be accessed here:

http://www.biomedcentral.com/1471-230X/9/63/prepub
